# Torsional Behaviour of Steel Fibre Reinforced Alkali Activated Concrete

**DOI:** 10.3390/ma13153423

**Published:** 2020-08-03

**Authors:** Chee Keong Lau, Trevor N. S. Htut, Jack J. Melling, Amin Chegenizadeh, Tian Sing Ng

**Affiliations:** 1 School of Civil and Mechanical Engineering, Curtin University, Kent Street, Bentley, WA 6102, Australia; amin.chegenizadeh@curtin.edu.au; 2 ETTOL Engineered Solutions, 1/41 Catalano Circuit, Canning Vale, WA 6155, Australia; trevor.htut@ettol.com.au (T.N.S.H.); jack.melling@ettol.com.au (J.J.M.); 3 JASMAT Steel Fabrications, 11 Baldwin Street, Kewdale, WA 6105, Australia; 4 PRASCON, Suite 1A, Level 2, 802 Pacific Highway, Gordon, Sydney, NSW 2072, Australia; tian@prascon.com

**Keywords:** alkali activated concrete, torsion, steel fibres, geopolymer, fly ash, ground granulated blast furnace slag

## Abstract

Nine alkali-activated concrete beams were produced and tested under pure torsional load to failure. The alkali-activated concrete beams were produced with following variables: (i) fibres only, (ii) conventionally reinforced or (iii) a hybrid of both fibres and conventional steel reinforcement. The fibres only beams were found to have approximately 20% higher cracking torque than conventionally reinforced beams. However, fibres only beams were observed to have lower post crack ductility and inconsistent post crack behaviour, in comparison to conventionally reinforced alkali-activated concrete (AAC) beams. On the other hand, the hybrid reinforcements in AAC beams were found to demonstrate more ductile post crack behaviour consistently of the beams tested. Hybrid reinforcement was also shown to have 20% and 25% improvement in cracking and ultimate torque compared to conventionally reinforced, which suggests that it is suitable for industrial applications to improve structure capacity. The ultimate torque results of the beams were compared to an analytical model that considered the contribution of fibres. It was found that the ultimate torque of the hybrid reinforced beam has good correlation with the model but overestimated conventionally reinforced beams.

## 1. Introduction

Concrete is the second most used material in the world after water, with concrete being applied in a large proportion of built environment including homes, roads, railways, skyscrapers, bridges and dams [1,2]. The production of ordinary Portland cement (OPC) used in concrete production emits a significant amount of CO_2_ where it could have the potential to emit 0.81 kg of CO_2_ for every kg of cement produced [3]. Alkali activated materials (AAM) (also known as geopolymer) holds the tremendous potential for reducing the CO_2_ emission of the construction industry and is often regarded as a greener option to OPC [4]. The implementation of waste material from steel and coal power stations, ground granulated blast furnace slag (GGBFS) and fly ash (FA) in alkali-activated concrete (AAC) might be able to reduce 40–80% of Portland cement associated emissions [5]. In addition to its potential environmental benefits, AAM also offers similar or superior properties in rapid strength gain, high-performance applications, chemical resistance and high-temperature stability [6,7,8,9,10]. 

The addition of fibres in the concrete matrix has been used since ancient civilisations such as Babylonians, Romans or Egyptians, with straws, horsehair and other forms of fibres added into their concrete-like composite materials. One particular example is the Romans adding horsehair into their mixes to reduce shrinkage, suggesting that addition of fibres in a binder-matrix material is not a modern invention [11]. In the last 50 years, the interest in fibres reinforced concrete has heightened after Batson et al. (1972) [12] demonstrated that shear reinforcements can be replaced with fibres in the mortar matrix, introducing a new potential tool for structural design. Since then, fibre reinforced concrete has been a major research area due to the ability of fibres in crack bridging, which provides additional capacity to structural members. The addition of steel fibre has been proven to be beneficial for OPC concrete beams under forces as the presence of fibres improves concrete’s poor performance in tensile [13]. However, it is important to point out that the fibres do not provide tensile resistance until the concrete matrix is cracked to engage the fibre–matrix bond. The tensile strength of the cracked fibre reinforced concrete is largely provided mechanically through the friction of fibre–matrix and also the fibre anchorage [14]. The additional tensile strength by fibres in concrete is also able to prevent sudden brittle failure occurring after a crack propagates through the matrix while it transforms the brittle failure mode into a pseudo ductile failure [13]. 

**Figure 1:**
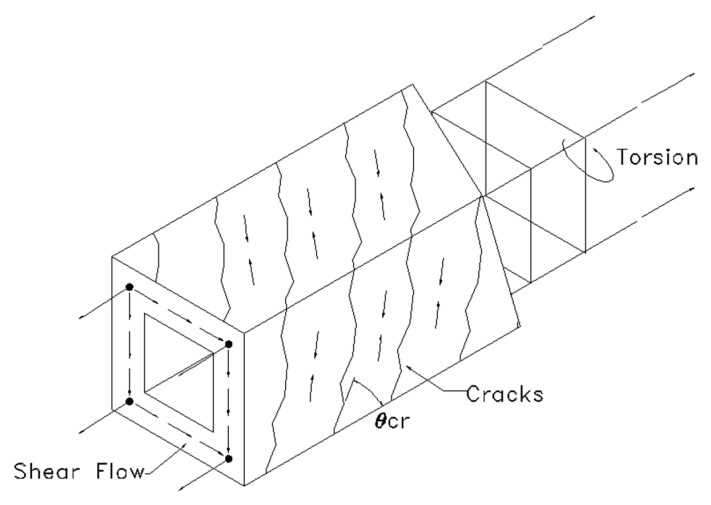
Space truss idealisation of beams in torsion.

Torsion is generally considered in the design of beams that are eccentrically loaded, curved beams or box girders for bridges, in which an eccentric load exerts a twisting moment on the section that leads to rapid crack propagation that is wider and less unsightly than flexural cracks [15]. It is common to idealise beams in torsion only as a space truss with the torsional action largely resisted by the compressive diagonal truss of the concrete with the tensile strengths of both transverse and longitudinal steel reinforcements [16]. The addition of fibres in beams under torsional forces would change the mechanism considerably as the tensile capacity of fibres bridging the crack are able to reduce the strain of the steel reinforcements [15]. Beams in torsion is commonly idealised as a space truss that consists of compressive concrete struts inclines at an angle *θ_v_*, with the system in equilibrium with the longitudinal and transverse bars in tension [15,17,18]. The space truss idealisation is shown in Figure 1.

Consequently, it is known that the application of fibre reinforced concrete for structural members in torsion leads to up to 60% increase in torsional capacity [19,20]. The addition of fibres in concrete also brings supplementary benefits such as the reduction in concrete cover spalling and decrease in crack width and crack spacing; a decrease in deflection can be easily attained with fibre reinforced concrete beams in torsion [19,21,22,23]. Additionally, Ju et al. (2019)’s recent work had proposed that the addition of fibres in concrete can be used as a minimum torsional reinforcement in place of traditional stirrups; thus, fibre reinforced concrete can be a useful workaround for steel congested members in torsion [18]. This further highlights the benefits and suitability of fibre reinforced concrete applications in the industry. 

Several recent research works have been conducted on pure and fibre reinforced AAC performance under torsional forces [24,25,26,27]. To the author’s knowledge, there is also no study conducted on the post crack performance of purely fibre reinforced beams in torsional load to compare with conventional and hybrid reinforcements of both fibres and steel reinforcements. This paper presents the behaviour of steel fibres in different combinations: fibres only, conventional reinforcements or hybrid of both. The experimental data collected are also compared with an existing analytical torsional capacity model based on Modified Field Compression Field Theory (MCFT) [28]. 

## 2. Experimental Investigation

### 2.1. Materials

All beams produced in this study are from alkali-activated concrete, with 56 MPa compressive strength on average. The main composition of the alkali-activated concrete is: Fly Ash (FA), silica fumes (SF), ground granulated blast furnace slag (GGBFS), steel fibres, alkali activators and aggregates consist of river sand and crushed gravel. 

The FA was sourced from Gladstone coal power plants in New South Wales, Australia. It was used as the primary aluminosilicate material for the production of the alkali-activated concrete. In accordance with ASTM C618, the FA is classified as Class F with calcium content is below 10% [29]. To enable ambient setting and improved compressive strength of the alkali-activated concrete, commercially available GGBFS from Westbuild was added at 15% of the total binder content. The specific gravities of the FA and GGBFS were 2.4 and 3.0. The mix design used in this study was derived from the previous study with the amorphous calcium content reduced to ensure sufficient setting time for casting [30]. SF was also added to ensure that the concrete strength to exceed 50 MPa for the optimal fibre–matrix anchorage of the fibres. The specific gravity of the silica fumes used was 2.2. The bulk oxide composition properties of the aluminosilicate material used in this study were obtained via X-ray Fluorescence analysis of the GGBFS, FA and SF and are shown in Table 1.

For the alkali activators, sodium hydroxide (NaOH) powder and sodium silicate (Na_2_SiO_3_) solution were used as the alkaline activator solution. The sodium hydroxide (97–98% purity) fine powder was prepared by dissolving in water to produce 12 M aqueous sodium hydroxide solution with 480 g of the alkali powder in each litre of potable tap water. Meanwhile, the D-grade sodium silicate with a sodium oxide to silicon dioxide molar ratio (MR) of 1.6 < MR ≤ 2.6 also used. The ratio of sodium hydroxide to sodium silicate was maintained at 2.5 by weight for all mixes to ensure optimal strength and workability. The aggregates used in the production of the beams consisted of natural sand as fine aggregate and crushed gravel as coarse aggregate with a nominal maximum size of 1.18 mm and 7 mm, respectively. The mix design of the AAC concrete is summarised in Table 2.

The double end hooked Dramix 5D 65/60BG steel fibres, provided by BOSFA Australia, were used in this study. The diameter and the length of the fibres were 0.9 mm and 60 mm. The ultimate tensile strength of the fibres was 2300 N/mm^2^. The amount of the steel fibres added to the mix was kept at the constant 0.5% volume fraction for all fibre reinforced beams. 

### 2.2. Specimens Details

**Figure 2:**
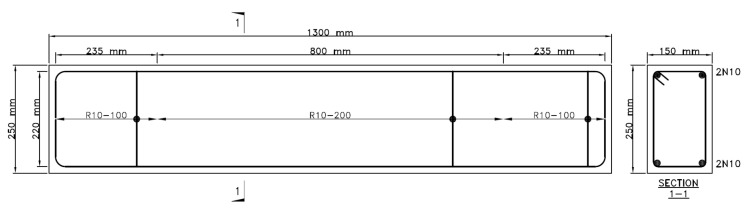
Details of torsion beam reinforcement. The measurements are shown in millimetres. R10-200 indicates Type R bars with 10 mm diameter at 200 mm spacing.

Three series of AAC beams were produced to investigate the contribution of fibres to conventional steel reinforced beams. The beams are designated with the F, R and FR notation to represent the beams that are fibre reinforced only (F), conventional reinforcement (R) and a hybrid reinforcement of fibres and conventional reinforcements (FR). For each series, three specimens were produced. The dimension of each beam was 250 mm in depth, 150 mm in width and 1300 mm in length with 15 mm concrete cover. As the beams were designed to be failed by torsion, additional reinforcement was provided at the supports to prevent crushing at the supports due to localised failure and produce a weak point at the midspan, allowing skew torsional failure to occur there. Sufficient anchorage of longitudinal steel was provided by extending the cog ends beyond the centre line of the loading arms. This was done to ensure the thin hollow tube space truss interaction to occur [15,31]. The Australian Type R with 250 MPa was used as closed-looped transverse stirrups reinforcement while the Type N bars with 500 MPa yield strength were used as longitudinal bars in this study. The mandrel diameter of the stirrups complied with the requirements of the Australian concrete design code (AS3600) where the mandrel diameter is at least 4 times of the bar diameter [32]. Both bars were 10 mm in diameter. The details of the beam are shown in Figure 2.

### 2.3. Specimen Preparation, Mixing and Curing

Sample preparation began with the aggregates moved from the laboratory aggregates pits, transferred to an oven, and dried at 105 °C for at least 24 h to remove the water content from the fine and coarse aggregates. Then, the oven-dried aggregates were removed from the oven and cooled to room temperature before specimen casting. Before casting, the moulds were cleaned and greased to ensure the ease of removal before mixing as AAC tends to be more difficult to remove compared to OPC. 

The production of the beams was conducted with a 70-L pan mixer to mix the AAC. The aluminosilicate materials (FA and silica fumes), a small amount of potable water and the aggregate were first mixed for 5 min, with both sodium hydroxide and sodium silicate mixed in a separate container as the material was mixed in the pan mixer. After 5 min, the alkaline activator mixture added to the dry mix for a further 5 min of mixing. Then, GGBFS was added gradually subsequently into the wet mix to prevent the flash setting of fresh AAC and ensure sufficient workability [33]. The fresh AAC was allowed to mix for a further 3 min with the remaining potable water added, which was followed by casting of the compressive and tensile strength cylinders with the fresh AAC. No steel fibres were added at this point; the presence of steel fibres in the characteristic testing cylinders would not produce representative results for both tests as steel fibres affect the strut and tie mechanism of the cylinders during testing [34]. Additionally, the fibres only have contribution after the concrete is cracked with the residual tensile strength [35]. Therefore, the steel fibres were added to the fresh AAC mix uniformly in the pan mixer after the cylinders were cast for an additional 5 min to prevent balling of steel fibres and allow even distribution of fibres in the fresh mix. Finally, the fresh AAC concrete was poured into the beam formwork on a casting bed and compacted with vibrator probes. After the pour, the beam specimen was covered with wet Hessian sheets and plastic sheets to prevent moisture loss until demoulding. The formwork of the beams was removed about 24 h after casting. Then, the specimen wrapped in pallet wraps to reduce the loss of the moisture on the surface of the beam and moved into the steam room for optimal curing at 70 °C for 24 h. 

### 2.4. Experimental Program

#### 2.4.1. Torsion Test Setup

**Figure 3:**
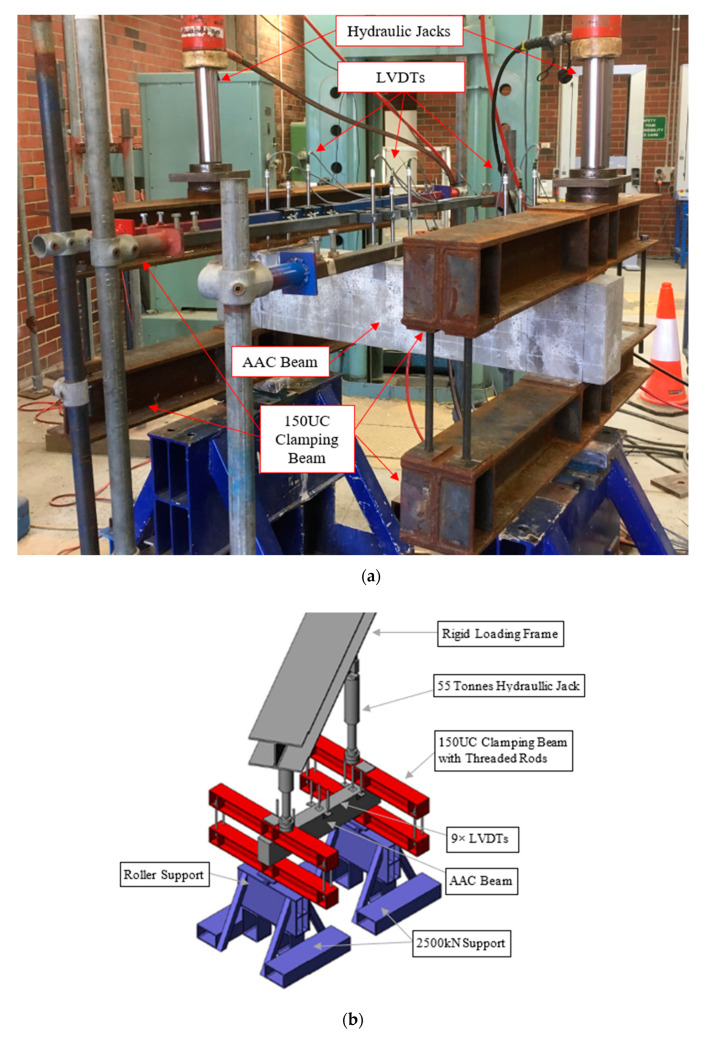
Torsion testing setup: (**a**) photograph of the setup; and (**b**) isometric view in 3D (the hollow section beams supporting the LVDT is not shown in the 3D sketch).

The test setup for torsional testing is shown in Figure 3a,b. The system adopts the principle with the application of two Universal Columns (UC) structural steel beams to clamp the AAC beams with 4 threaded rods. The rotation force by two hydraulic jacks was simultaneously applied with a constant 200-mm lever arm from the centre of the AAC beam to produce torsional moments. The torsional moments were exerted at a loading rate of 0.1 mm per minute. The hydraulic jacks were mounted on a stiff loading frame through an attached load cell. The UC section was supported by a pinned support that consisted of a rod welded to a steel plate and a wedged plate to allow rotational movement during the test. Nine linear variable differential transducers (LVDT) were used to measure the vertical movement along the top of the beam. The centre row of the LVDT was used as a reference point to calculate the rotation of the ends of the beam under torsional forces. There was the possibility of cracks forming under the LDVT with the data collected from the LDVT positioned on cracks removed during analysis. The data acquisition system (DAS) was utilised to record the forces on the load cells and the displacement of the LDVTs. 

Nine beams were tested under torsional forces applied at 200 mm from the centre of the beam. Beam specimens produced with steel fibres only are denoted as beam specimens F1, F2 and F3. R1, R2 and R3 are used to denote the beams produced with solely conventional reinforcement. Finally, the hybrid combination of both steel fibres and conventional reinforcements are designated as RF1, RF2 and RF3. 

#### 2.4.2. Compressive Strength

The compressive strength of the unreinforced AAC was tested following AS 1012.9:2014 [36]. The loading rate of the compressive strength was controlled at 20 MPa/min. The specimens for the test had a dimension of 100 mm in diameter and 200 mm in height. 

#### 2.4.3. Indirect Tensile Strength

Indirect tensile strength of the unreinforced AAC was conducted per AS 1012.10:2000 (R2014) [37]. The size of each specimen was 150 mm in diameter and 300 mm in height. 

#### 2.4.4. Crack Mouth Opening Displacement (CMOD) 

Steel fibres reinforced AAC beams of 150 mm by 150 mm by 550 mm were produced to measure the post crack performance of the concrete. A 25 mm notch was cut on the mid-span of the beam. The CMOD test was conducted in accordance with EN 14651 (2005) to investigate the residual tensile strength performance of the fibre [38]. 

## 3. Results and Discussion

### 3.1. Material Properties

The results of the average compressive strength, tensile strength and CMOD are summarised in Table 3. BOSFA Australia recommends that the minimum concrete compressive strength of 50 MPa achieves optimal performance of the anchorage of the double end hooked 5D fibres. In this study, the average compressive strength was found to be 56 MPa. The compressive strength obtained shows that the AAC matrix in this study can fully utilise the fibre’s anchorage. This is also supported by the post cracking tensile performance obtained, which was found to be 3.0 MPa.

### 3.2. The Crack Pattern on AAC Beams

The crack patterns of all the fibre only beams were found to be consistent among the three beams with a single spiralling crack localised on the beam. The average angle for the compressive struts of the AAC beams, *θ_v_*, was 50° on average for AAC beams, which is higher than the 45° in the space truss theory [17]. The *θ_v_* recorded in this study was also higher than the value observed by Van Mier (2013), with 35–40° for the unconfined torsional test by Mode III loading of concrete and mortar cylinders [39]. In accordance with AS3600, *θ_v_* was calculated to be 40.5° using the General Method (Clause 8.2.4.2) [32]. The MFCT equations for OPC beams under pure torsion derived by Amin and Bentz estimated the *θ_v_* angle as 43° [15]. It is important to note that most design standards and models are derived based on the well-understood properties of OPC. Hence, it was hypothesised AAC may have noticeable differences in the material properties to OPC at the meso level, which may lead to higher *θ_v_* angles. Table 4 shows a summary of the compressive strut angles, *θ_v_.*

The angle *θ_v_* was approximately 52° for the fibre only beams. No spalling of AAC was observed among the three beams tested as presence fibres in the concrete matrix could prevent spalling because of the ability to transfer tensile stress over a crack formed [15]. During the testing of the fibre only beams, it was observed that the single localised crack was formed in a brittle and abrupt manner after the concrete was cracked, with a loud creaking sound of the fibres being engaged. Then, it was followed by significantly increased rotation and a drop in torque recorded, in which the tests of this batch had to be stopped early due to significant crack widening, a rapid increase in the angle of rotation and safety concerns. No other cracks were formed or diverged from the major crack. This is likely because the longitudinal chords from the space truss model were not present due to the absence of longitudinal reinforcements in the beam that led to the inability to transfer some stress from torsional moments, with the stresses from torsional forces carried by the fibre reinforced AAC. Interestingly, vertical splitting cracks were also observed on the AAC beams near the support of the beams This occurred due to the lack of longitudinal ties in the fibres only AAC beams, which led to flexural or shear failure. The spiral crack formed on the fibres only beams were incomplete where the spiral crack did not extend to both sides of the beams. According to Van Meir (2013), the cross-section area of the beams was reduced with the occurrence of spiral crack and the remaining intact cross-section might not have sufficient capacity to carry the confining axial stress [39]. Hence, this led to a vertical splitting crack that is similar to Mode II loading. However, a complete brittle failure of the AAC beams was avoided as the steel fibres used in this study provided limited ductility after the first crack occurred. 

**Figure 4:**
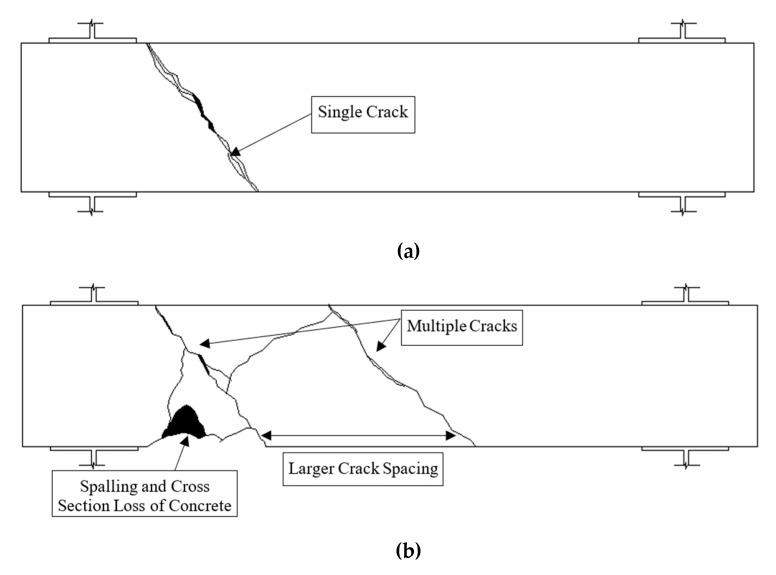

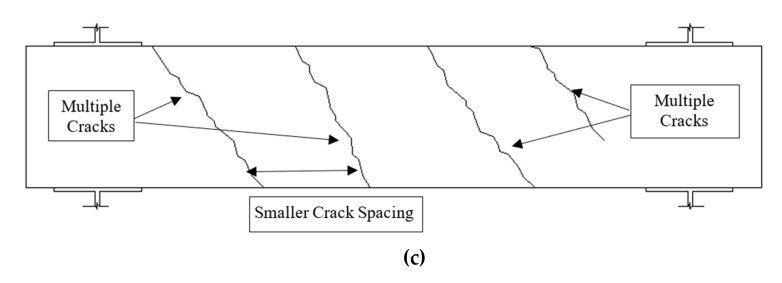
Cracking patterns based on reinforcement types: (**a**) fibre only reinforcement; (**b**) conventional reinforcement only; and, (**c**) hybrid fibre and conventional reinforcement.

**Figure 5:**
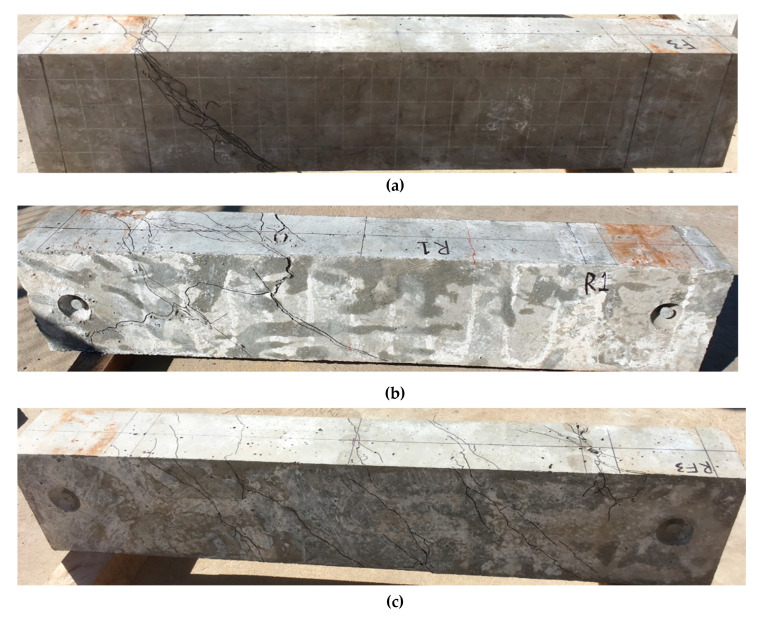
Photographs of the crack patterns based on reinforcement types: (**a**) fibre only reinforcement; (**b**) conventional reinforcement only; and, (**c**) hybrid fibre and conventional reinforcement.

Multiple diagonal compressive strut cracks were observed on the conventionally reinforced AAC beams in this scenario, which is supportive evidence of the space truss theory also being valid on AAC material. The average *θ_v_* of this series of beams was 49°, about 10% higher than the assumed 45° in earlier studies [17]. It is important to note that the cracking behaviour of conventional reinforced series differed from the fibre only series in terms of its cracking behaviour. The conventional reinforcement AAC beams were not observed to display the same abrupt formation of localised crack after the first crack as purely fibre reinforced beams due to the presence of the longitudinal and transverse chords to provide some robustness post crack. The three tested beams displayed more post crack ductile behaviour in comparison, with diverged cracks also found on the conventionally reinforced beams as opposed to localised cracks in purely fibre reinforced beams. However, the low tensile capacity of AAC led to spalling on the beams for the conventionally reinforced beams, which, in turn, led to the loss of beam cross-section during the tests. The observation of spalling in this study at a high angle of rotation is consistent with the literature [13,21]. The findings are presented in Figure 4 and Figure 5. 

With a hybrid reinforcement of steel reinforcement and fibres, the developed spiral cracks were narrower and more closely spaced, as indicated in Figure 4 and Figure 5. The crack width of most cracks formed was also smaller than conventionally reinforced. The formation of cracks in this series of beams was also gradual compared to the sudden formation of the purely fibre reinforced beams. No spalling was observed with hybrid reinforcements of all three beams. This was due to the increased tensile strength of the AAC matrix, which allowed the AAC to redistribute tensile strength over the cracks to other regions of the beam with higher crack surface area, to provide additional robustness to beams after the first crack. With the ability to increase tensile capacity over cracks, spalling of AAC was also prevented. The observations in this study are consistent with conventionally and fibre reinforced OPC beams in the literature [13,15,19]. The post crack behaviour of these beams was also the most ductile among the series of beams tested in this study. Interestingly, the average *θ_v_* was fairly consistent with conventionally reinforced and pure fibre reinforcements. The *θ_v_* angle of hybrid reinforcements was 51°.

### 3.3. Behaviour and Strength of AAC Beams under Torsion

*T_cr_* is the torque exerted on the beam when the first visible torsional crack occurred or first drop in torque during testing, and *T_u_* is the maximum torsional load recorded throughout the torsional test. The *T_cr_* and *T_u_* of each beam coincide with the respective angle of rotation at the first crack (*θ_cr_*) and ultimate torque (*θ_u_*). 

**Figure 6:**
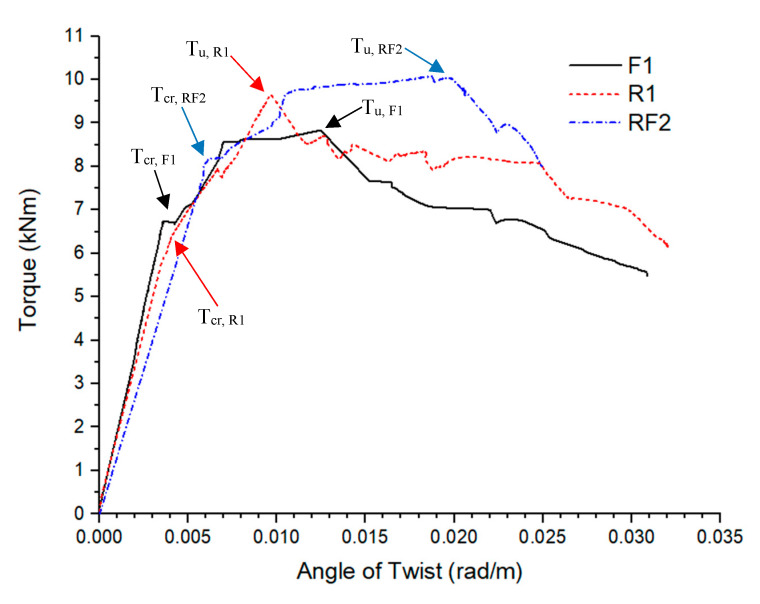
Comparison of the torque versus angle of rotation of purely fibre reinforcement, conventional reinforcement and the hybrid reinforcement of both fibres and conventional reinforcements.

The torque and angle of rotation values of conventionally reinforced AAC beams are designated as a reference to the other two series. The average *T_cr_* and *T_u_* of conventionally reinforced AAC beams were 6.3 kNm and 8.3 kNm at average *θ_cr_* of 0.0031 rad/m and *θ_u_* of 0.0058. Figure 6 illustrates the plots of torque versus angle of rotation of AAC beams produced with fibres only, conventional reinforcement and the hybrid reinforcement of both.

**Figure 7:**
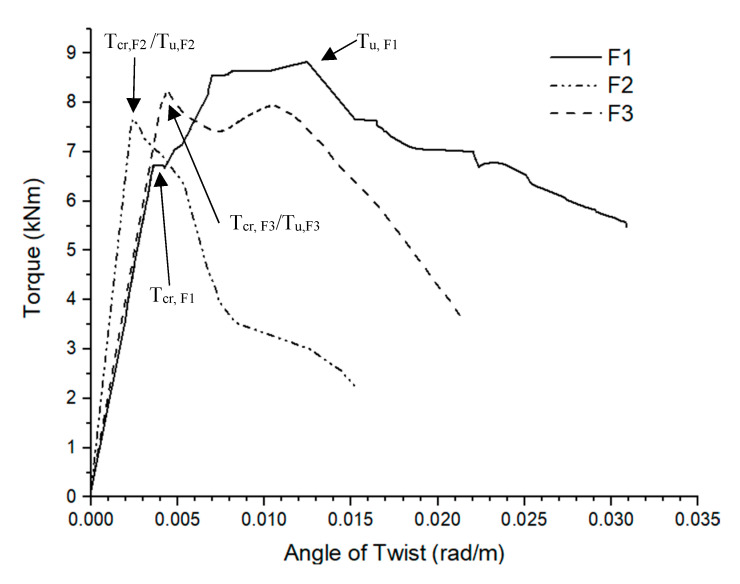
Comparison of the torque vs. angle of twist for beam specimens produced with fibre reinforcement only.

For the fibres only reinforced beam series, the average *T_cr_* and *T_u_* were 7.5 kNm and 8.3 kNm at average *θ_cr_* of 0.0035 rad/m and *θ_u_* of 0.0065 rad/m. The *T_u_* of fibre reinforced beams were similar to conventionally reinforced beams in torsion but the *T_cr_* of fibre reinforced beams were about 20% higher than conventionally reinforced beams. The average angle of rotation between fibre reinforced and conventionally was also observed to be similar in the first crack and ultimate crack scenario. The torsional performance of solely fibre reinforced AAC beams was found to have comparable performance to conventionally reinforced AAC beams until the first crack, despite the absence of any of the steel bars in the fibres only beams. In contrast, the post crack ductility after *θ_u_* was comparably poorer than conventional reinforcements, suggesting that AAC beams in torsion still require conventional steel reinforcements for consistent and sufficient post crack ductility to ensure the progressive collapse of structural members. Based on the torque versus angle of twist relationship shown in Figure 7, there was a range of behaviours observed in the fibre only specimens. 

**Figure 8:**
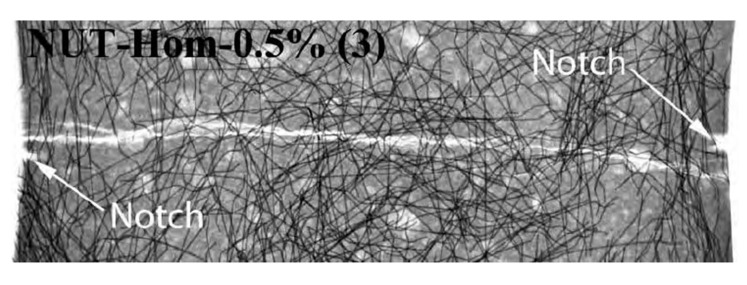
X-ray imaging of steel fibre reinforced OPC at 0.5% volume fraction of dog bone specimens showing the crack path was influenced by fibre dispersion with crack propagating through the section of concrete with the least resistance [41].

Figure 7 shows that F1 and F3 displayed considerably improved strain-softening behaviour post crack while F2 showed a comparably less ductile behaviour instead. However, the use of steel fibres avoided sudden brittle failure of AAC beams under torsion. The behaviour was inconsistent among the beams tested, with only two out of three beams displaying the improved post crack strain-softening behaviour. The reason of the variation in post crack performance of the three fibre only AAC beams tested was because the OPC optimised double end-hooked geometry of the fibre anchorage might not be best suited for AAC applications as the strong bearing forces from high adhesion strength of AAC might lead to earlier brittle failure of the matrix, followed by softening post crack behaviour [7,40]. No fibre pullouts were observed during testing. The volume fraction percentage at 0.5% of steel fibre utilised in the AAC mixes could suggest another reason for the issue of inconsistent post crack behaviour due to fibre dispersion. Htut (2010) reported that cracks often initiate at regions of the fibre–matrix with the lowest amount of fibre concentration [41]. Cracks tend to propagate through the path of least resistance in the fibre–matrix, particularly with fibres added at low volume fraction. According to Figure 8, the observed cracks tended to travel around the path of least resistance, which was around the end hooks of the fibre without engaging the fibre at sections of a fibre reinforced concrete with a low volume fraction of fibres. It is also important to note that, at a higher volume fraction of fibre addition, the occurrence of cracks propagating along the path of least resistance would be less likely to occur. It is believed that a similar crack mechanism occurred with the fibres only AAC beams in torsion that lead to inconsistent post cracking behaviour.

The absence of the longitudinal and transverse cords provided by the space truss model could be the reason for the lower angle of rotation of fibres only AAC beams, in comparison to beams that has conventional reinforcements. Based on space truss idealisation of beams in torsion, the torsional strength of the beams is expressed by the yield stress of the longitudinal and transverse steel as well as the geometry of the reinforcing cage while the tensile strength of concrete does not contribute directly to torsion capacity in an unreinforced matrix. It is important to note that the conventional analysis in design codes generally assumes concrete does not have a tensile contribution for torsion. In contrast, for the pure fibre reinforced AAC beams, the post-peak ductility was contributed purely by the residual tensile strength of the steel fibres to avoid brittle failure. Although with the less ductile post-peak softening response of pure fibre reinforced to conventional reinforcements, the contribution of the fibres in AAC beams to post crack ductility could be considered significant, as an unreinforced AAC beam would have failed with brittle failure after first crack [13]. 

For hybrid reinforcement beams with both steel fibres and conventional reinforcement, the average *T_cr_* and *T_u_* of hybrid reinforcements beams were 7.5 kNm and 10.5 kNm. The average angle of rotation of the hybrid reinforcements was indicated with *θ_cr_* of 0.0046 rad/m and *θ_u_* of 0.0158 rad/m, respectively. The application of both reinforcement types concurrently was found to have significantly improved torsional strength of AAC beam, particularly from the angle of rotation for first crack and ultimate torque. With reference to conventionally reinforced AAC beams, the *θ_cr_* and *θ_u_* in hybrid reinforcement beams were found 50% and 170% higher than the angle of rotations recorded for solely conventional reinforcements in AAC beams. The peak torque of hybrid reinforcements, *T_u_*, was also found to have increased 26% over conventional reinforcements. There was slight increase of approximately 20% in *T_cr_* of hybrid reinforced AAC beams. Therefore, the performance of hybrid fibres in AAC beams displays significant benefits with the addition of steel fibres to conventionally reinforced AAC beams. This can be explained as the addition of steel fibres enables the AAC to provide tensile capacity by the crack bridging mechanism to a beam under torsion. As cracks formed due to combined mixed-mode fracture on the concrete beam, the steel fibres were engaged to bridge the crack, thus allowing the stresses to transfer over the crack [42]. With the fibres engaged after cracking, the stress within the beam would transfer to the uncracked areas of the beam with less fibre content, where microcracking would occur until single crack began to widen. This process will repeat until the failure of the beam. This leads to formations of multiple compression strut cracks to dissipate some of the torsional forces. Hence, the hybrid reinforcement of steel fibres and conventional reinforcements in AAC beams increases the torsional strength of the beams. The findings of the beneficiation of steel fibres addition in concrete under torsional load are consistent with the literature [20,21,27].

The beams produced with hybrid fibres also displayed improved post crack strain-softening behaviour consistently across the beam specimens tested, in comparison to the fibres only beams. It can be suggested that the conventional steel reinforcement in hybrid reinforcements allows the geometry reinforcement cage to function as a torsional restraint in space truss to provide more robustness in the post-crack performance. Then, the steel fibres work in conjunction with conventional steel to redistribute the tensile stresses along with the hollow tube idealisation of the space truss in torsion near the surface of the beam to prevent localised cracks, as observed in conventional steel only batches. Particularly, the increased *T_cr_* torque was flagged in this study with the beams that have steel fibre reinforcements. With the absence of the conventional reinforcements, the fibre only beams in this study showed inconsistent strain-softening post crack due to the occurrence of cracks in AAC matrix tend to nucleate and propagate through a pathway with the least amount of fibres [43,44]. However, the presence of conventional reinforcements in hybrid reinforcements seemed to suggest that the inconsistent post crack behaviour of fibre only beams can be prevented with better post crack softening behaviour occurring more consistently in hybrid reinforced beams. Hence, the application of hybrid fibres allows the torsional capacity of AAC beams to be increased along with improved post crack ductility with consistent post crack behaviour. The *T_cr_*, *T_u_*, *θ_cr_* and *θ_u_* experimental results are tabulated in Table 5. However, the data for RF1 are not presented here due to an unexpected technical issue occurring in the data logging system.

### 3.4. Toughness Index 

The ASTM C1018-97 is adapted for analysis of beams after the first crack for investigation on the behaviour of the beams under torsion [45]. The analysis aims to characterise the behaviour of the fibre reinforced beams after the onset of the first crack based on fixed points of the deflection to calculate the toughness of the beam. It is also to overcome the shortcoming of first-crack strength assessments as the contribution of fibres may not be a significant improvement of first crack performance to non-fibre reinforced concrete [45]. ASTM C1018 is designed for fibre reinforced beams under flexural with the deflection of the mid-span of the beam measured. Hence, in this scenario, the angle of rotation of the beam in torsion was replaced the deflection of δ, 3δ, 5.5δ and 10.5δ. δ is originally denoted in ASTM C1018-97. The same was applied for the deflection when the first crack occurred with the notation denoted as the angle of rotation at the first crack (*θ_cr_*) for AAC beams in torsion. 

The toughness index, *I_5_, I*_10_ and *I*_20_, is calculated with the corresponding area under the curve at 3δ, 5.5δ and 10.5δ from origin to δ. The toughness indices allow the comparison of the beams post crack performance in torsion after first crack at 3, 5.5 or 10 times of the angle of rotation of the initial crack. The purpose of adopting the principles from the ASTM C1019-97 is to allow comparisons between the three types of reinforcements in the AAC beams at different stages of the post crack curves. Concerning the torque versus angle of twist curves, the toughness index of *I_5_, I*_10_ and *I*_20_ were calculated with Equations (1)–(3), as per ASTM C1018-97 [45].

(1) $$I_{5}=\frac{Area\;under\;the\;curve\;from\;origin\;to\;3\delta }{Area\;under\;the\;curve\;from\;origin\;to\;\delta }$$

(2) $$I_{10}=\frac{Area\;under\;the\;curve\;from\;origin\;to\;5.5\delta }{Area\;under\;the\;curve\;from\;origin\;to\;\delta }$$

(3) $$I_{20}=\frac{Area\;under\;the\;curve\;from\;origin\;to\;10.5\delta }{Area\;under\;the\;curve\;from\;origin\;to\;\delta }$$

Based on ASTM C1018, the residual strength factors of *R*_5,10_ and *R*_10,20_ can be derived from the toughness indices above to quantify the percentage of average torque retained after cracking over a specific deflection to the first crack load. The residual strength factors of *R*_5,10_ and *R*_10,20_ can be defined as Equations (4) and (5) [45]:

(4) $$R_{5,10}=20\times \left(I_{10}-I_{5}\right)$$

(5) $$R_{10,20}=10\times \left(I_{20}-I_{10}\right)$$

**Figure 9:**
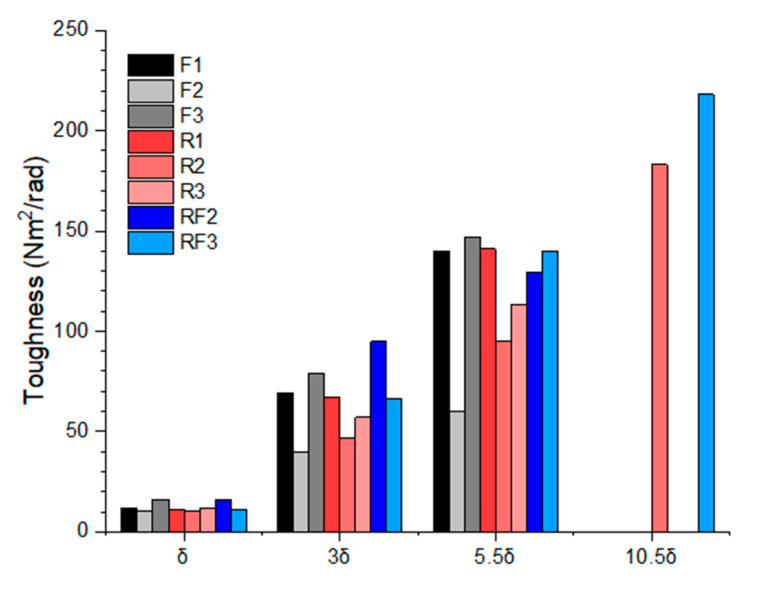
The plot of the area under the curve of torque versus angle of twist at δ, 3δ, 5.5δ and 10.5δ.

**Figure 10:**
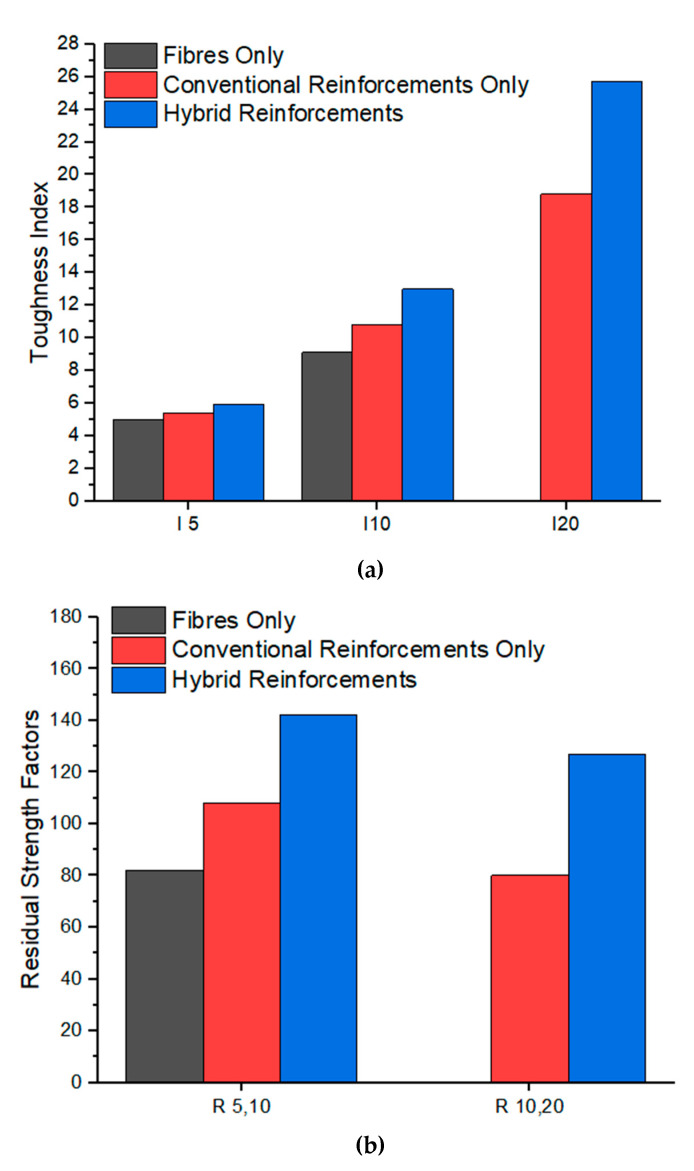
Toughness indices (**a**) and residual strength factors (**b**) of AAC beams in torsion

It should be noted that there are no data available for fibres only AAC beams as the test had to be terminated after the 5.5δ point due to excessive rotation and large crack width where these beams were considered to reach failure. The crack width of the fibres only AAC beams in the series were exceeding the quarter length of the fibres, where it is known as the complete failure of the fibre [35]. The calculated area under the graph is shown in Table 6 and Figure 9. The average toughness index and residual strength index of eight beams tested are plotted in Table 7 and Figure 10, respectively.

For the AAC beams with conventional reinforcements, the toughness indices shown in Table 7 and Figure 10 were used as a baseline for the comparison between the fibres only beams and hybrid reinforcements beams. Referring to the residual toughness factors of conventional reinforcements, it can be seen that there was slight hardening observed for *R*_5,10_, indicating the steel bars yielded. This was followed by a softening curve at *R*_10,20_, indicating a 20% drop in average load to the first crack. 

It can be noted that the fibres only beams were observed to have lower toughness indices at *I*_5_ and *I*_10_ to conventionally reinforced AAC beams, with its toughness indices lower by approximately 7% and 16%, respectively. This suggests that the pure fibres AAC beams did not offer similar torsional strength as conventional reinforcements. The lack of data of *I*_20_ for the fibres only beams is further evidence that pure fibre reinforcement offers a lower level of post crack ductility. In contrast to conventional reinforcements, the fibres only beams were found to record 18% drop at *R*_5,10_, showing the post-crack softening occurs earlier. Hence, fibres only AAC beams are unsuitable for industrial applications due to lower ductility to conventionally reinforced beams where beams in torsion should perform on par or better than conventionally reinforced ones [18]. 

On the other hand, the hybrid reinforcement of both conventional steel bars and the steel fibres showed noticeable improvements over conventionally reinforced AAC beams. In accordance with Figure 10, the hybrid reinforcement demonstrated percentage increased 10%, 20% and 36% compared to conventionally reinforced beams, with reference to the *I*_5_, *I*_10_ and *I*_20_ indices, respectively. *R*_5,10_ and *R*_10,20_ factors of the hybrid reinforcements also showed 142% and 127%, where the steel fibres addition in AAC could achieve higher ultimate torque and sustain the increased torsional resistance for a higher angle of twist. Furthermore, *R*_5,10_ and *R*_10,20_ factors were also approximately 34% and 47% higher than conventionally reinforced AAC beams. This further supports the addition of steel fibres to conventional steel can increase the post-crack torsional capacity, improving the angle of twist. 

### 3.5. Analytical Results

Amin and Bentz (2018) proposed a model for concrete beams under torsional load that is suitable for design, as demonstrated in Equation (6) [15]:

(6) $$T_{ua}=2A_{o}\times \min\left[\begin{array}{c} \left(\frac{A_{sv}f_{sv}}{s}+k_{fd}t_{c}f_{w}\right)cot\theta_{v}\;,\\ \;\left(\frac{\Sigma A_{sl,i}f_{sy,i}}{p}+k_{fd}t_{c}f_{w}\right)tan\theta_{v} \end{array}\right]\leq 0.25f_{cm}\frac{1.7A_{g}^{2}}{p}$$

where *A_o_* is the area enclosed by the centreline of shear flow path; *s*, *A_sv_* and *f_sv_* are the spacing, area (one leg only) and yield strength of transverse reinforcement; *A_sl_* and *f_sy_* are the area and yield strength of each longitudinal steel bar; *p* is the perimeter of the concrete section (gross); *k_fd_* is the fibre dispersion reduction factor, set as 0.82 to consider the effect of cracks propagating through the sections with the lowest number of fibres in the concrete matrix [43,44]; *f_w_* is the residual tensile strength, calculated in accordance to AS3600-2018 [32]; *θ_v_* is the principal compressive stress or the strut angle; *f_cm_* is the average compressive strength of unreinforced AAC cylinders; *A_g_* is the gross cross-section of the AAC beam; and *t_c_* is the equivalent hollow tube section thickness [15], calculated as:

(7) $$t_{c}=0.75\frac{A_{g}}{p}$$

Based on the simplified Modified Compression Field Theory (MCFT), the compressive strut angle, *θ_v_*, can be calculated by iterating Equations (8)–(10) [28]. *E_s_* is the elastic modulus of steel reinforcements with *A_st_* and *A_sb_* the areas of the top and bottom longitudinal steel.

(8) $$\theta_{v}=29{}^{\circ}+7000\varepsilon_{x}$$

(9) $$\varepsilon_{w}=\frac{0.9T_{ua}}{2A_{o}E_{s}\left(A_{sB}+A_{sT}\right)}$$

(10) $$w=0.2+1000\varepsilon_{x}\geq 0.125\;mm$$

Amin and Bentz (2018) proposed a non-iterative version of the model that is more suitable for design in the industry [15]. The non-iterative variant of the equation sets the strain of the concrete beam at mid-height to 80% of the yield strain for the bottom layer of the steel reinforcement, with *ε_x_ =* 0.8*f_sy_/E_s_*. The modified *θ_v_* equation is demonstrated as Equation (11) [15]:

(11) $$\theta_{v}=29{}^{\circ}+0.028f_{sy}$$

Table 8 summarises the analytical ultimate torque, *T_ua_*, calculated with Equations (6) and (11). Regarding AS3600-2018, *T_us_* of the conventionally reinforced AAC beams is also calculated for further comparison as the current code does not allow the design of concrete beams in torsion with fibres [32]. The ratio of *T_u_* to *T_ua_* and *T_u_* to *T_us_* of each beam tested was calculated for corroboration between the experimental ultimate torque and analytical results, as shown in Table 8.

Based on Table 8, the Amin and Bentz (2018) model demonstrated a good correlation to the experimental *T_u_* of hybrid conventional and fibre reinforced beams, with an average *T_u_/T_ua_* ratio of 1.12 and a small coefficient of variance of 0.05. The conventional reinforced AAC beams showed a higher value of average *T_u_/T_ua_* ratio of 1.71 with a higher coefficient of variance of 0.15 with Amin and Bentz model. However, the current AS3600 concrete design code indicates the average ratio of *T_u_/T_us_* is lower at 1.21 with the same coefficient of variance at 0.15. The values indicate the steel reinforcements contribution of beams in torsion is conservative, as AAC is known to display up to 10% increased bond strength of steel reinforcements compared to OPC [6]. For the fibres only AAC beams, the average *T_u_/T_ua_* ratio is significantly higher at 2.36, suggesting that the fibre contribution of Amin and Bentz (2017) [15] model may be underestimated for the steel fibre contributions. Further research is required to increase the model accuracy and the suitability of the model for AAC. 

## 4. Conclusions

This paper presents the investigation in the torsional strength of three series of AAC beams with different types of reinforcements. Different behaviours of AAC beams under torsional loadings were successfully demonstrated. The following conclusions could be reached from this study:

1. In terms of the practicality for industry usage, this study recommended that steel reinforcements are still required for concrete beams in torsion. Steel fibres should be used to compliment steel reinforcement bars for the increased torsional capacity of concrete beams and improved post-crack ductility as the application of steel fibres in unreinforced concrete beams does not provide sufficient ductility after cracking at 0.5% volume fraction.
2. The behaviour of AAC beams under torsion was found to be similar to OPC. The inclusion of steel fibres in conventional reinforced AAC is able to effectively control spalling, decreased crack width, increased torsional resistance and the angle of twist and improved post-crack ductility over conventionally reinforced AAC beams. The hybrid reinforcement demonstrated higher toughness indices over conventional reinforcements, which further supports the application of steel fibres in concrete beams.
3. The investigation of fibres only beams under torsion could prevent sudden brittle failure of pure AAC beams in torsion with increased post-crack ductility. It was also found that *T_cr_* of the fibres only beams were higher than conventionally reinforced due to the contribution of fibres to delay the onset of cracks by fibres’ crack bridging mechanism. The *I*_5_ and *I*_10_ indices of fibres only AAC beams were lower than those of conventionally reinforced AAC beams. However, the post-crack ductility observed in this study was not comparable to conventionally reinforced beams. Therefore, the application of fibres only beams in torsion in industry application is not recommended based on the observations in this study.
4. The simplified MCFT based analytical model was applied to the experimental results. It was found that the model could predict the torsional strength of the AAC beams with a reasonable correlation of experimental to the analytical ratio of 1.12 and coefficient of variance of 0.07. However, it underestimated the torsional strength of AAC beams with conventional reinforcements while the AS3600 (2019) estimates were closer to this study’s experimental torsional strength values. Additional testing is highly recommended to further examine the adequacy of the model.

## Figures and Tables

**Table 1:** Bulk oxide composition (wt %) of FA, GGBFS and SF used in this study with the LOI at 1000 °C from XRF analysis.

Elemental Oxide	GGBFS	FA	SF
Al_2_O_3_	13.2	22.8	0.5
CaO	40.9	6.8	1.0
Fe_2_O_3_	0.9	3.8	1.2
K_2_O	0.3	1.3	1.8
MgO	5.4	1.3	1.9
MnO	0.2	0.1	0.1
Na_2_O	0.1	0.1	0.7
SO_3_	4.9	0.3	0.5
SiO_2_	33.2	60.0	89.8
TiO_2_	0.6	1.1	0.1
LOI (1000 °C)	0.3	1.2	3.0

**Table 2:** Mix Design of the AAC (kg/m^3^).

FA	GGBFS	SF	NaOH	Na_2_SiO_3_	Additional Water	Coarse Aggregate	Fine Aggregate
290	56	26	57	143	17	1102	705

**Table 3:** Average compressive strength, tensile strength and residual tensile strength of the AAC concrete.

Average Compressive Strength (MPa)		Average Tensile Strength (MPa)	CMOD with Notched 3 Points Bending Test (MPa)				
f_cm_	f′_c_	f_ct_	f_R1_	f_R2_	f_R3_	f_R4_	f_w_
67.0	56.4	5.6	6.7	7.8	8.9	9.8	3.0

**Table 4:** θ_v_ of each beam tested under torsional forces.

	Fibres Reinforcement Only			Conventional Reinforcement Only			Hybrid Reinforcement		
**Beam**	F1	F2	F3	R1	R2	R3	RF1	RF2	RF3
**Strut Angle (°)**	53	53	50	50	50	48	48	53	52
**Average Angle (°)**	52			49			51		

**Table 5:** Experimental results from each beam tested under torsional forces.

	T_cr_, (kNm)	θ_cr_ (rad/m)	T_u_, (kNm)	θ_u_ (rad/m)	T_u_/T_cr_
**F1**	6.7	0.0038	8.8	0.0125	1.3
**F2**	7.7	0.0026	7.7	0.0026	1.0
**F3**	8.0	0.0041	8.3	0.0044	1.1
**R1**	5.7	0.0033	9.8	0.0103	1.7
**R2**	5.8	0.0027	7.6	0.0035	1.3
**R3**	7.5	0.0032	7.7	0.0036	1.1
**RF2**	7.2	0.0058	10.1	0.0193	1.4
**RF3**	7.8	0.0034	10.8	0.0122	1.4

**Table 6:** The area under the curve of torque versus angle of twist (Nm^2^/rad) at δ, 3δ, 5.5δ and 10.5δ.

	F1	F2	F3	R1	R2	R3	RF2	RF3
**δ**	12	10	16	11	10	12	16	11
**3δ**	69	40	79	67	47	57	95	66
**5.5δ**	140	60	147	141	95	113	129	140
**10.5δ**	N/A	N/A	N/A	N/A	183	N/A	N/A	218

**Table 7:** Average toughness indices and residual strength factors of the beams in torsion.

	I_5_	I_10_	I_20_	R_5,10_ (%)	R_10,20_ (%)
**Fibres Only**	5	9	N/A	82	N/A
**Conventional Reinforcements Only**	5	11	19	108	80
**Hybrid of Fibres and Conventional Reinforcements**	6	13	26	142	127

**Table 8:** Torsional strength comparison of experimental results to MCFT and AS3600 analytical results. Brackets indicate the coefficient of variance (CoV) of the average ratio of experimental torque to analytical.

	Experimental	MCFT Model		Average T_u_/T_ua_ Ratio (CoV)	AS3600		Average T_u_/T_us_ Ratio (CoV)
	T_u_ (kNm)	T_ua_ (kNm)	T_u_/T_ua_		T_us_ (kNm)	T_u_/T_us_	
**F1**	8.8	3.50	2.51	2.36 (0.07)	-	-	-
**F2**	7.7	3.50	2.20		-	-	
**F3**	8.3	3.50	2.37		-	-	
**R1**	9.8	4.89	2.00	1.71 (0.15)	6.91	1.42	1.21 (0.15)
**R2**	7.6	4.89	1.55		6.91	1.10	
**R3**	7.7	4.89	1.57		6.91	1.11	
**RF2**	10.1	9.36	1.08	1.12 (0.05)	-	-	-
**RF3**	10.8	9.36	1.15		-	-	

## References

[1] Scrivener K.L., John V.M., Gartner E.M. (2018). Eco-efficient cements: Potential economically viable solutions for a low-CO_2_ cement-based materials industry. Cem. Concr. Res..

[2] Hendriks C.A., Worell E., De Jager D., Blok K., Riemer P. Emission Reduction of Greenhouse Gases from the Cement Industry. Proceedings of the Fourth International Conference on Greenhouse Gas Controlled Technologies.

[3] Heath A., Paine K., McManus M. (2014). Minimising the global warming potential of clay based geopolymers. J. Clean. Prod..

[4] Li N., Shi C., Zhang Z., Wang H., Liu Y. (2019). A review on mixture design methods for geopolymer concrete. Compos. Part B Eng..

[5] Habert G., Ouellet-Plamondon C. (2016). Recent update on the environmental impact of geopolymers. RILEM Tech. Lett..

[6] Castel A., Foster S.J. (2015). Bond strength between blended slag and Class F fly ash geopolymer concrete with steel reinforcement. Cem. Concr. Res..

[7] Liu Y., Shi C., Zhang Z., Li N., Shi D. (2020). Mechanical and fracture properties of ultra-high performance geopolymer concrete: Effects of steel fiber and silica fume. Cem. Concr. Compos..

[8] Zhang J., Shi C., Zhang Z., Ou Z. (2017). Durability of alkali-activated materials in aggressive environments: A review on recent studies. Constr. Build. Mater..

[9] Barbosa V.F.F., MacKenzie K.J.D. (2003). Thermal behaviour of inorganic geopolymers and composites derived from sodium polysialate. Mater. Res. Bull..

[10] Davidovits J. (2008). Geopolymer Chemistry and Applications.

[11] Domone P., Illston J. (2010). Construction Materials: Their Nature and Behavior.

[12] Batson G., Jenkins E., Spatney R. (1972). Steel fibers as shear reinforcement in beams. J. Proc..

[13] Chalioris C.E., Karayannis C.G. (2009). Effectiveness of the use of steel fibres on the torsional behaviour of flanged concrete beams. Cem. Concr. Compos..

[14] Löfgren I. (2005). Fibre-Reinforced Concrete For Industrial Construction. Ph.D. Thesis.

[15] Amin A., Bentz E.C. (2017). Strength of steel fiber reinforced concrete beams in pure torsion. Struct. Concr..

[16] Collins M.P., Mitchell D. (1991). Prestressed Concrete Structures.

[17] Rausch E. (1929). Design of Reinforced Concrete for Torsion and Shear (Berechnung des Eisenbetons gegen Verdrehung und Abscheren).

[18] Ju H., Lee D.H., Kim K.S. (2019). Minimum torsional reinforcement ratio for reinforced concrete members with steel fibers. Compos. Struct..

[19] Narayanan R., Kareem-Palanjian A.S. (1986). Torsion in Beams Reinforced with Bars and Fibers. J. Struct. Eng..

[20] Ju H., Kim K.S., Lee D.H., Hwang J.H., Choi S.H., Oh Y. (2015). Torsional responses of steel fiber-reinforced concrete members. Compos. Struct..

[21] Facconi L., Minelli F., Plizzari G., Ceresa P. Experimental study on steel fiber reinforced concrete beams in pure torsion. In Proceeding of the fib Symposium in Krakow.

[22] Mansur M.A., Paramasivam P. (1982). Steel fibre reinforced concrete beams in pure torsion. Int. J. Cem. Compos. Lightweight Concr..

[23] Patil S.P., Sangle K.K. (2016). Tests of steel fibre reinforced concrete beams under predominant torsion. J. Build. Eng..

[24] Aulia T.B., Muttaqin M., Afifuddin M., Zaki M., Nastiti G. (2019). Effect of using geopolymer flyash on torsion capacity of hybrid high-strength reinforced concrete beams containing fine and coarse aggregates substitution which added iron ores as filler. J. Phys. Conf. Ser..

[25] Kumar M. (2017). Experimental Study On The Behaviour Of Torsion In Geopolymer Concrete Beams With Steel Fibers. J. Ind. Pollut. Control.

[26] Prachasaree W., Abideng H. (2017). Prediction of Torsional Strength for Very High Early Strength Geopolymer. Mater. Sci..

[27] Daniel A.J., Sivakamasundari S., Nishanth A. (2017). Study on Partial Replacement of Silica Fume Based Geopolymer Concrete Beam Behavior under Torsion. Proc. Eng..

[28] Bentz E.C., Vecchio F.J., Collins M.P. (2006). Simplified modified compression field theory for calculating shear strength of reinforced concrete elements. ACI Mater. J..

[29] (2017). ASTM International, ASTM C618-17a -Standard Specification for Coal Fly Ash and Raw or Calcined Natural Pozzolan for Use in Concrete.

[30] Lau C.K., Rowles M.R., Parnham G.N., Htut T., Ng T.S. (2019). Investigation of geopolymers containing fly ash and ground-granulated blast-furnace slag blended by amorphous ratios. Constr. Build. Mater..

[31] Gu X., Jin X., Zhou Y. (2016). Torsion. Basic Principles of Concrete Structures.

[32] (2019). Standards Australia, AS3600 - Concrete Structures.

[33] Ng T.S., Amin A., Foster S.J. (2013). The behaviour of steel-fibre-reinforced geopolymer concrete beams in shear. Mag. Concr. Res..

[34] Olesen J.F., Østergaard L., Stang H. (2006). Nonlinear fracture mechanics and plasticity of the split cylinder test. Mater. Struct..

[35] Ng T.S., Htut T., Foster S.J. (May 2012). Fracture of Steel Fibre Reinforced Concrete - The Unified Variable Engagement Model.

[36] Standards Australia (2014). AS 1012.9:2014-Methods of testing concrete. Method 9: Compressive Strength Tests—Concrete, Mortar and Grout Specimens.

[37] Standards Australia (2014). AS 1012.10-2014-Methods of Testing Concrete Determination of Indirect Tensile Strength of Concrete Cylinders.

[38] European Committee for Standardization (2005). Test Method for Metallic Fibered Concrete—Measuring the Flexural Tensile Strength (Limit of Proportionality (LOP), Residual).

[39] Mier J.G.M.V. (2013). Concrete Fracture: A Multiscale Approach.

[40] Bhutta A., Farooq M., Zanotti C., Banthia N. (2016). Pull-out behavior of different fibers in geopolymer mortars: Effects of alkaline solution concentration and curing. Mater. Struct..

[41] Htut T. (2010). Fracture Processes In Steel Fibre Reinforced Concrete. Ph.D. Thesis.

[42] Mier J.G.M.V. (1997). Fracture Processes of Concrete: Assesements of Material Parameter for Fracture Models.

[43] Foster S., Htut T., Ng T. (2013). High performance fibre reinforced concrete: Fundamental behaviour and modelling. Proceedings of the 8th International Conference on Fracture Mechanics of Concrete and Concrete Structures, FraMCoS 2013.

[44] Amin A. (2015). Post Cracking Behaviour Of Steel Fibre Reinforced Concrete: From Material To Structure. Ph.D. Thesis.

[45] ASTM International (1997). C1018-97: Standard Test Method for Flexural Toughness and First-Crack Strength of Fiber-Reinforced Concrete (Using Beam With Third-Point Loading).

